# Cerebral Ischemia Increases Small Ubiquitin-Like Modifier Conjugation within Human Penumbral Tissue: Radiological–Pathological Correlation

**DOI:** 10.3389/fneur.2017.00738

**Published:** 2018-01-12

**Authors:** Joshua D. Bernstock, Daniel G. Ye, Allison Griffin, Yang-ja Lee, John Lynch, Lawrence L. Latour, Gregory K. Friedman, Dragan Maric, John M. Hallenbeck

**Affiliations:** ^1^Stroke Branch, National Institute of Neurological Disorders and Stroke, National Institutes of Health, Bethesda, MD, United States; ^2^Department of Clinical Neurosciences, Wellcome Trust-Medical Research Council Stem Cell Institute, University of Cambridge, Cambridge, United Kingdom; ^3^Section on Stroke Diagnostics and Therapeutics, National Institute of Neurological Disorders and Stroke, National Institutes of Health, Bethesda, MD, United States; ^4^Center for Neuroscience and Regenerative Medicine at the Uniformed Services University of the Health Sciences, Bethesda, MD, United States; ^5^Division of Pediatric Hematology and Oncology, Department of Pediatrics, University of Alabama at Birmingham, Birmingham, AL, United States; ^6^Flow and Imaging Cytometry Core Facility, National Institute of Neurological Disorders and Stroke, National Institutes of Health, Bethesda, MD, United States

**Keywords:** SUMOylation, ischemic stroke, neuroprotection, magnetic resonance imaging, penumbra

## Abstract

Posttranslational modification by small ubiquitin-like modifier (SUMO) regulates myriad physiological processes within cells and has been demonstrated to be highly activated in murine brains after cerebral ischemia. Numerous *in vitro* and murine *in vivo* studies have demonstrated that this increased SUMO conjugation is an endogenous neuroprotective stress response that has potential in being leveraged to develop novel therapies for ischemic stroke. However, SUMO activation has not yet been studied in poststroke human brains, presenting a clear limitation in translating experimental successes in murine models to human patients. Accordingly, here, we present a case wherein the brain tissue of a stroke patient (procured shortly after death) was processed by multiplex immunohistochemistry to investigate SUMO activation.

## Introduction

**Figure 1:**
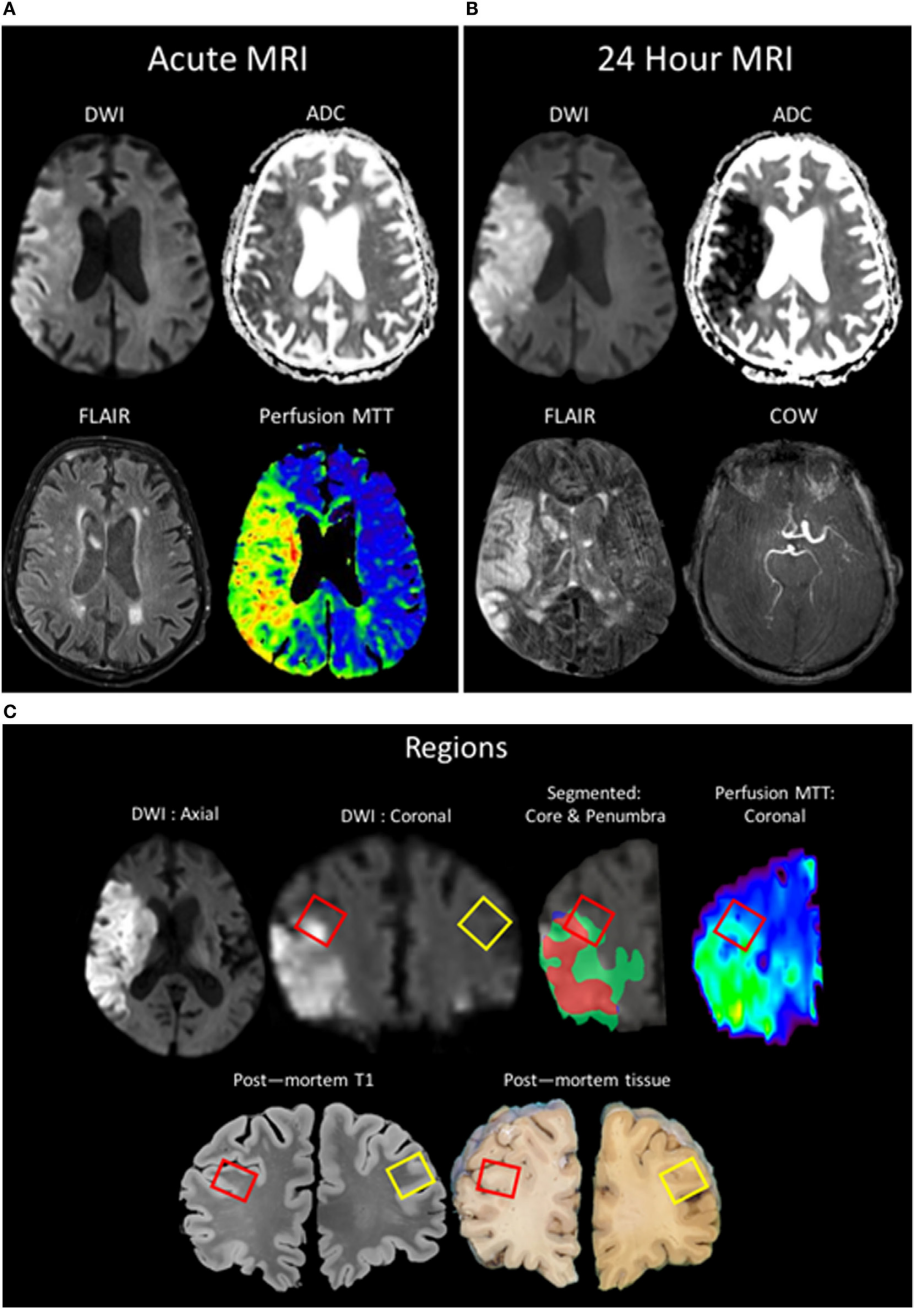
Patient MR images taken during clinical evaluation (acute) **(A)** and 24 h later **(B)** [pictured: trace-weighted diffusion weighted imaging (DWI) and ADC maps, fluid-attenuated inversion recovery (FLAIR), perfusion mean-transit time (MTT), and a minimum intensity projection of the circle of willis]. **(C)** MR images taken postmortem were analyzed to co-localize regions of interest identified *in vivo* with the postmortem sample (red boxes: ipsilateral ROI; yellow boxes: contralateral). Two regions of interest were segmented, the region of the core (red) taken from the baseline DWI and the region of the penumbra as the mismatch between the MTT and the DWI (green), and overlaid onto the 24-h DWI.

An 89-year-old man presented to our facility with acute onset of left-sided weakness and hemineglect. Magnetic resonance imaging (MRI) revealed a right middle cerebral artery (MCA) stroke with associated hypoperfusion of the right MCA territory and a thrombus in the distal right internal carotid artery. The patient was treated with intravenous recombinant tissue plasminogen activator (rtPA). The patient’s family provided surrogate consent, and the patient was enrolled in an IRB-approved natural history observational study (ClinicalTrials.gov NCT00009243). The patient worsened neurologically over the following 24 h and was placed on comfort care. The patient expired 43 h after hospital admission due to cardiorespiratory failure. The family provided written consent for an unrestricted donation for diagnostic, scientific, or therapeutic purposes, and an autopsy was performed within 12 h of death. Brain tissue was obtained at autopsy and stored at the National Cancer Institute Laboratory of Pathology. The surfaces of the cerebrum and brainstem showed mild atrophy, but no softening or masses. There was no evidence of herniation of the cingulate gyri, unci, or cerebellar tonsils. Postmortem MRI of the brain was obtained after fixation at 7T and co-registered to the *in vivo* MR images obtained at stroke onset. Brain samples for immunofluorescent staining were derived from the right frontal lobe at the periphery of the ischemic region within an area of hypoperfusion as confirmed by MRI, as well as from the contralateral region of the left frontal lobe; the penumbra was characterized as areas visualized as normal for diffusion but abnormal for perfusion (Figure 1).

## Background and Discussion

Posttranslational modification by small ubiquitin-like modifier (SUMO) regulates diverse homeostatic processes within cells (1). After the demonstration of SUMO activation during hibernation torpor (2), further experiments have shown that both global and focal transient cerebral ischemia–reperfusion dramatically increase the levels and nuclear localization of SUMO-conjugated proteins within murine brains, and furthermore, this process may contribute toward neuroprotection (3–9, 19). Consequently, numerous studies in murine models have sought to induce protection against ischemia by leveraging SUMOylation (10–12). However, at present, evidence of SUMO activation in postischemic human brain tissue has yet to be demonstrated.

**Figure 2:**
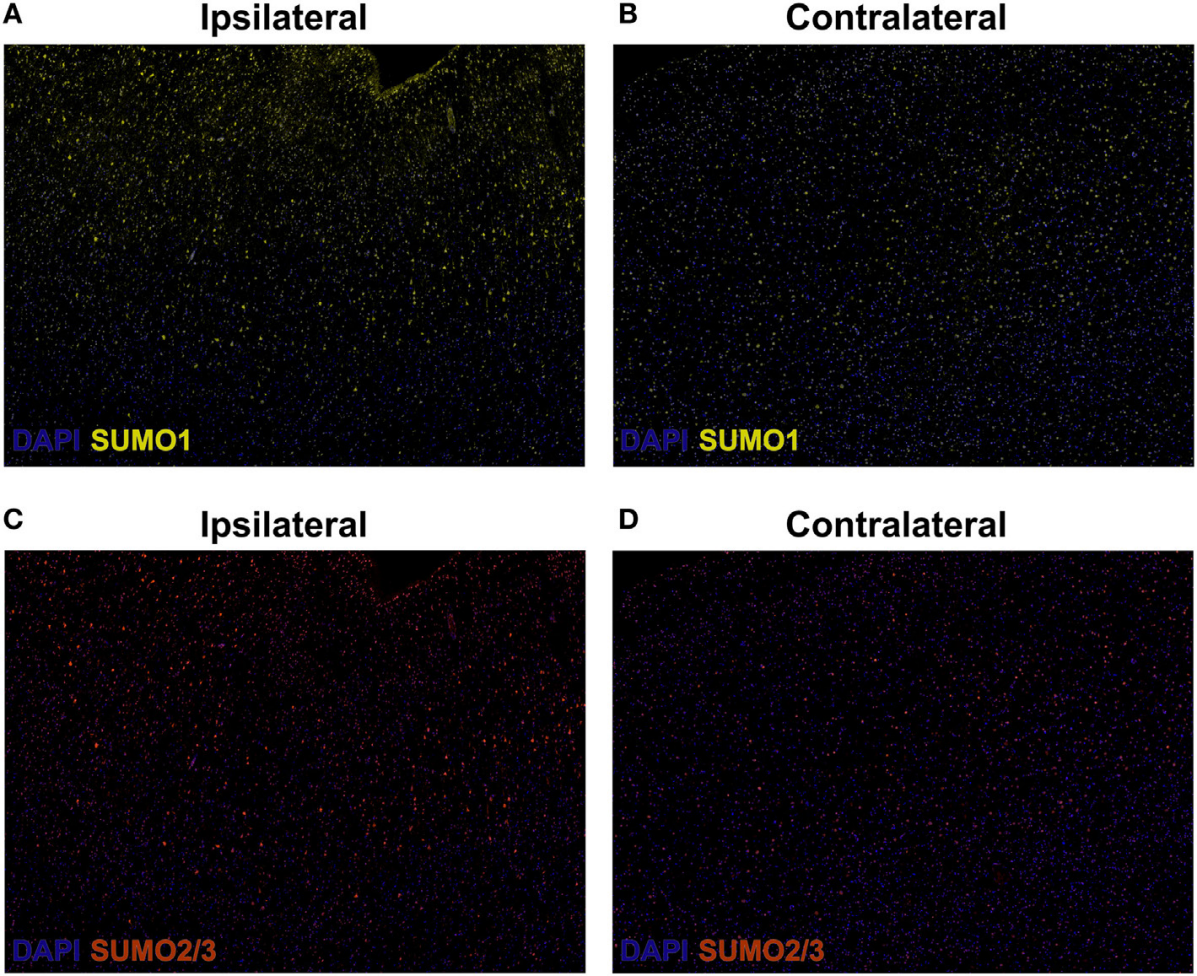
Composite wide-field fluorescent multiplex immunohistochemistry (mIHC) images (yellow, SUMO1; red, SUMO2/3; blue, DAPI,). **(A)** Ipsilateral, SUMO1. **(B)** Contralateral, SUMO1. **(C)** Ipsilateral, SUMO2/3. **(D)** Contralateral, SUMO2/3. Capture parameters of ipsilateral and contralateral images were identical. Cropped ROIs were taken from comparable layers of cortex. The intensity of SUMO1 and SUMO2/3 immunoreactivity is increased in neurons residing within the penumbral tissue compared to neurons in the matched contralateral anatomy.

**Figure 3:**
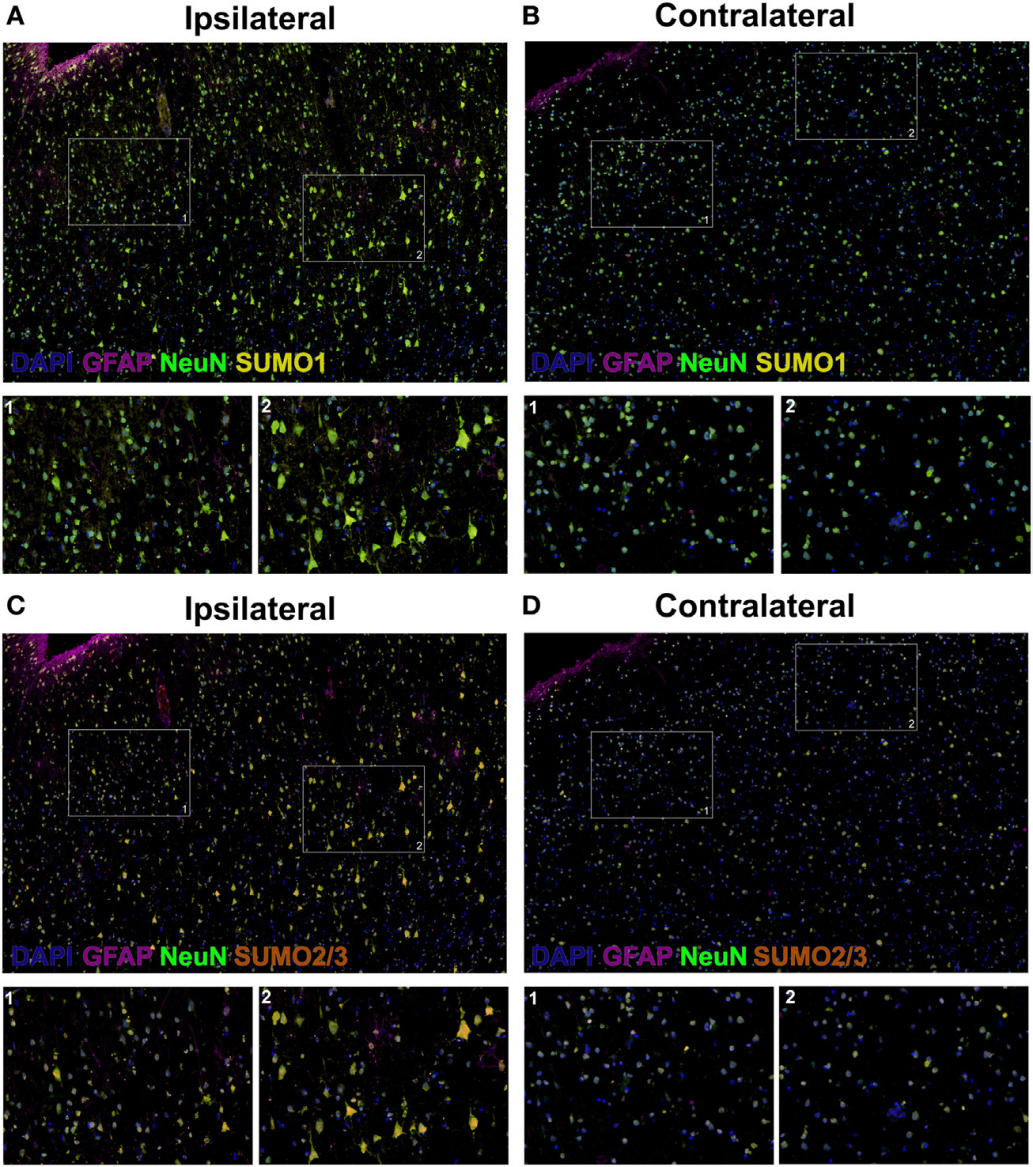
High-resolution composite fluorescent multiplex immunohistochemistry (mIHC) images (yellow, SUMO1; red, SUMO2/3; blue, DAPI; green, NeuN; violet, GFAP). **(A)** Ipsilateral, SUMO1. **(B)** Contralateral, SUMO1. **(C)** Ipsilateral, SUMO2/3. **(D)** Contralateral, SUMO2/3. Capture parameters of ipsilateral and contralateral images were identical. Cropped ROIs were taken from comparable layers of cortex. The intensity of SUMO1 and SUMO2/3 immunoreactivity is increased in neurons residing within the penumbral tissue compared to neurons in the matched contralateral anatomy; in addition, SUMO1 and SUMO2/3 immunoreactivity is translated from the cytoplasm to the nucleus in penumbral neuronal tissue.

Herein, fluorescent multiplex immunohistochemistry (mIHC) was employed to visualize the intensity and localization of SUMO within the neurons of the penumbral tissue and the corresponding tissue of the contralateral hemisphere. Upregulation of SUMO1 and SUMO2/3 in neurons of the ischemic penumbra was clearly observed in the form of an increased intensity of immunoreactivity compared to the matched contralateral tissue (Figure 2). Furthermore, SUMO1 and SUMO2/3 immunoreactivity was also observed to translocate from the cytoplasm to the nucleus (visualized with DAPI) in greater intensity in penumbral neurons compared to contralateral neurons (Figure 3).

Briefly, after an ischemic event, the affected tissue can be divided into three regions based on factors including collateral perfusion, susceptibility to cell death, and duration of blood vessel occlusion: the ischemic core, the ischemic penumbra, and the oligemia (13–16). The ischemic core is tissue that has suffered irreversible injury due to severe reductions in blood flow (<20% of normal) and oxygen delivery and is as such considered unsalvageable (15, 17). The ischemic penumbra comprises severely hypoperfused (~40% of normal) tissue, is functionally impaired, is progressively recruited into the ischemic core as the duration of vascular occlusion increases (being detectable with PET as late as 18 h after onset), and is considered salvageable if perfusion is restored (15, 17, 18). Thus, the ischemic penumbra is the target of acute reperfusion therapies (e.g., thrombolysis using rtPA), which improve patient outcomes in proportion to the volume of penumbra ultimately salvaged (18). Finally, the oligemia surrounds the penumbra and suffers a mildly reduced CBF (>40% of normal). Tissue in the oligemia is generally not at risk barring complications (15). Ultimately, as the ischemic penumbra is where therapeutic interventions in ischemic stroke are most relevant and efficacious (with the understanding that the SUMO pathway has been identified as a potential therapeutic target in animal models of stroke), and considering that SUMOylation in the brain is a rapidly cycling ATP-dependent process (1, 8), tissue from the ischemic penumbra was selected for mIHC.

Past studies in murine models of ischemia have demonstrated increased SUMO conjugation in the brain after ischemia–reperfusion. Notably, the greatest levels of SUMO activation and neuronal nuclear localization of SUMO2/3-conjugated proteins were observed in cells of the ischemic penumbra (4, 19). Significant research investigating the neuroprotective effect of SUMO activation in *in vitro* and murine models of ischemia have already been conducted, demonstrating the critical role of SUMO activation as an endogenous neuroprotective stress response that attenuates neuronal damage caused by ischemia–reperfusion, as well as enhanced neuroprotection when SUMO activation is induced above physiological levels (2, 5–7, 10, 20–22). Cells overexpressing the SUMO E2 conjugase, Ubc9, and subsequently demonstrating increased SUMO conjugation, were protected from oxygen/glucose deprivation (OGD)-induced damage, whereas cells with an inhibited Ubc9 demonstrated decreased SUMO conjugation and were sensitized to OGD-induced damage (2). The overexpression of SUMO1 and SUMO2 protected cells from OGD-induced damage, while microRNA-induced depletion of endogenous SUMO1 and SUMO2/3 sensitized cells to OGD-induced damage (5, 20). Suppressing SUMOylation through overexpression of SENP1 also sensitized cortical neurons to OGD-induced damage (21). Transgenic mice overexpressing Ubc9 had increased levels of SUMOylated proteins in their brains, were more tolerant to ischemic stress, and had smaller infarct volumes after pMCAO compared to wild-type mice (10). Meanwhile, transgenic SUMO-knockdown mice in which miRNAs specifically silenced SUMO1-3 in neurons displayed worse functional outcomes after transient forebrain ischemia compared to wild-type mice (9).

Naturally, as upregulation of the SUMOylation pathway has been shown to be neuroprotective in preclinical models of brain ischemia, several studies have sought to pharmacologically modify SUMOylation in an effort to bring this pathway to bear on acute ischemic insults (11, 12, 23). For example, a lead compound (N106) has been identified to increase SUMOylation *via* activation of the SUMO E1 enzyme and was capable of inducing protection against myocardial insults *in vitro* and *in vivo* (23). Other compounds such as histone deacetylase inhibitors and synthetic retinoids, which increase SUMOylation by inhibiting the regulatory miRNAs miR-182 and miR-183, have demonstrated protection of cortical neurons *in vitro* (11). After the discovery that quercetin is a potential SENP inhibitor that may induce neuroprotection in part *via* increased SUMOylation (12), quantitative high-throughput screens using physiologically relevant SUMO substrates have also been developed and employed to search for potential neuroprotective compounds that act through the inhibition of SENP2 (24). The topic of therapeutic strategies leveraging SUMOylation in brain ischemia has been recently reviewed in greater detail (25). Ultimately, the search for SUMO-activating compounds that may be developed into neuroprotective therapies in humans in ongoing.

## Concluding Remarks

To date, despite numerous animal studies and the clinical importance of developing neuroprotective therapies for ischemic stroke, there has been no evidence demonstrating SUMO activation in human brains after stroke. This report is the first to document that within a poststroke human brain, SUMOylation patterns are largely concordant with those in postischemic murine brains, suggesting that enhanced SUMO conjugation may play a similar role in humans as in murine models (i.e., as an endogenous neuroprotective stress response that could be therapeutically leveraged to attenuate ischemic damage and promote functional recovery). Thus, it is the authors’ contention that this case serves as an initial validation of the body of research into SUMO conjugation-induced neuroprotection, although it must be recognized that more studies should be conducted to confirm the generalizability of these results.

## Materials and Methods

### Magnetic Resonance Image Acquisition

As a part of the baseline clinical evaluation for stroke, and research follow-up, the subject was imaged on a 3T MR Scanner (Siemens Medical, Malvern, PA, USA) using a standardized MRI protocol that included diffusion-weighted imaging (DWI), T2* gradient recalled echo, time-of-flight magnetic resonance angiogram of the circle of willis, and T2-weighted fluid-attenuated inversion recovery (FLAIR). Relevant parameters for sequences presented are as follows: forty 3.5-mm thick axial-oblique slices aligned along the anterior–posterior commissure were acquired co-localized for DWI and FLAIR. DWI consisted of 15 direction tensor sequences used to generate both trace-weighted DWI and ADC maps, *b*-value = 1,000, TR/TE = 100,025x ms, FOV = 24 cm, and 3 mm × 3 mm × 7 mm voxels. FLAIR consisted of TR/TE/TI = 9,000/120/2,600 ms. Dynamic susceptibility contrast perfusion-weighted imaging was performed using an echo-planar T2*-weighed gradient recalled echo sequence with the relevant parameters: TR/TE = 1,200/25 ms, FA = 80, FOV = 220 mm, matrix of 96 × 96, twenty 7-mm axial-oblique slices, and 80 dynamics after a weight-adjusted single dose of Gd-BOPTA injected at 5 ml/s into the antecubital vein. Maps of mean-transit time were generated using vendor provided software with arterial input function deconvolution.

After extraction and 1-month fixation with 10% formalin, the brain was hydrated for 1 week in 1% formalin before being transferred to a custom-machined container attached to a 1/3-horsepower vacuum pump. The brain was then immersed under vacuum in fluorinated oil (Fomblin LC/8, Solvay Solexis Inc.) that was free of proton MR signal. Whole brain images were acquired using a 7T MRI scanner (Siemens, Erlangen, Germany) and analyzed to co-localize regions of interest that had been seen *in vivo* to the postmortem specimen. The brain was sectioned into coronal slices, and additional sectioning and histopathological processing was performed on ROIs and matching contralateral control tissues.

### Fluorescence mIHC

Briefly, 10-μm-thick human brain OCT-embedded sections were incubated with Human BD Fc Blocking solution (BD Biosciences) to block endogenous Fc receptors and then incubated in True Black Reagent (Biotium) to quench intrinsic tissue autofluorescence. The sections were then immunoreacted for 1 h at RT using 1–5 µg/ml cocktail mixture of the following immunocompatible primary antibodies: rat IgG2a anti-SUMO 1 (Sigma-Aldrich), mouse IgG1 anti-SUMO 2/3 (Abcam), mouse IgG2b anti-GFAP (BD Biosciences), and guinea pig IgG anti-NeuN (EMD Millipore). This step was followed by washing off excess primary antibodies with PBS supplemented with 1 mg/ml bovine serum albumin and staining the sections using a 1 µg/ml cocktail mixture of the appropriately cross-adsorbed secondary antibodies conjugated to one of the following spectrally compatible fluorophores (all purchased from Thermo Fisher): Alexa Fluor 488, Alexa Fluor 546, Alexa Fluor 594, and Alexa Fluor 647. After washing off excess secondary antibodies, sections were counterstained using 1 µg/ml DAPI (Thermo Fisher) for visualization of cell nuclei. Slides were then coverslipped using Immu-Mount medium (Thermo Fisher) and imaged using a multichannel wide-field epifluorescence microscope (see below).

### Fluorescence mIHC Image Acquisition

Images were acquired from whole specimen sections using the Axio Imager.Z2 slide scanning fluorescence microscope (Zeiss) equipped with a 20×/0.8 Plan-Apochromat (Phase-2) non-immersion objective (Zeiss), a high-resolution ORCA-Flash4.0 sCMOS digital camera (Hamamatsu), a 200 W X-Cite 200DC broad band lamp source (Excelitas Technologies), and five customized filter sets (Semrock) optimized to detect the following fluorophores: DAPI, Alexa Fluor 488, Alexa Fluor 546, Alexa Fluor 594, and Alexa Fluor 647. Image tiles (600 µm × 600 µm viewing area) were individually captured at 0.325 µm/pixel spatial resolution, and the tiles were seamlessly stitched into whole specimen images using the ZEN 2 image acquisition and analysis software program (Zeiss), with an appropriate color table having been applied to each image channel to either match its emission spectrum or to set a distinguishing color balance. Pseudocolored stitched images were then exported to Adobe Photoshop and overlaid as individual layers to create multicolored merged composites.

## Ethics Statement

This study was performed on postmortem human brain procured after the participant’s family provided written informed consent for an unrestricted autopsy. While living, the family gave written informed consent for the study as well as subsequent publication of medically relevant findings. The study (NCT00009243) was approved by the local Institutional Review Board.

## Author Contributions

All authors participated in the drafting/revision of the manuscript.

## Conflict of Interest Statement

This research was conducted in the absence of any commercial or financial relationships that could be construed as a potential conflict of interest.
